# Cancer Stem Cell Markers CD44, CD133 in Primary Gastric Adenocarcinoma

**Published:** 2014

**Authors:** Anahita Nosrati, Farshad Naghshvar, Somaieh Khanari

**Affiliations:** 1 *Department of Pathology,Imam Khomeini Hospital, Mazandaran University of Medical Sciences*, Sari, Iran.

**Keywords:** Primary gastric adenocarcinoma, cancer stem cell, CD44, CD133, immunohistochemistry

## Abstract

Cancer stem cells (CSCs) are unique subpopulations that have the capacity to drive malignant progression with renewal abilities. Recently the role of some of CSCs in gastric adenocarcinoma has been studied. This study was performed in order to evaluate CD44 and CD133 expressions by immunohistochemistry in 95 primary gastric adenocarcinoma and their relation to clinical and pathological parameters of these tumors. There was a significant correlation between CD44 expression and cancer subtype (intestinal), tumor size (4-8 cm), depth of invasion, no lymphatic/vascular invasion and moderate differentiation. There was a significant correlation between CD133 expression and patient's age (> 65 years), cancer subtype (intestinal), tumor size (4-8 cm), depth of invasion and moderate differentiation. CSC markers like CD 44 and CD133 had high expression in primary gastric adenocarcinoma and had some relations to clinical and pathological parameters of tumors.

Gastric cancer is the fourth most common malignancy and the second leading cause of cancer-related death for both sexes in the world (1, 2). Gastric cancer is the most common cancer within gastrointestinal tract in Iranian men (after esophagus and colorectal cancer) and the third most common cancer among Iranian women (after breast and colon cancer) (3.^4^).According to the report of Ministry of Health, it is the most common fetal malignancy of digestive tract and the most common cause of death due to cancer in Iran (5-7). The incidence of this cancer in Iran is twice of the global rate (4); furthermore, it is more common in the north and northwest of Iran (5). Most gastric cancers are adenocarcinomas, which according to pathological classifications, are divided into two types, intestinal and diffuse. Intestinal type usually occurs in older men, whereas, diffuse- type gastric adenocarcinoma tends to occur in young people and has the same frequency in males and females (8). Cancer stem cells (CSC) are a subgroup of tumor cells that have the capacity of self- renewal and multilineage differentiation (9, 10). The hypothesis of tumor stem cells was first established in hematological malignancies in 1997 and then were studied in many solid tumors (9). The cancer stem cells not only initiate tumor development, metastasis and recurrence, but also make routine treatment of cancer unsuccessful (10). To identify the gastric cancer cells, several studies have been conducted on some cell surface markers.

CD44 is a homing cell adhesion molecule, a cell surface glycoprotein for hyaluronic acid. This marker is expressed on the surface of red blood cells and platelets and they have been known as lymphocytic homing receptors. CD44 plays an important role in adhesion to the extracellular matrix and matrix functions, such as degeneration, proliferation and cell survival (2, 8, 10, 11). CD133 (prominin-1 gene) on chromosome 4p15 encodes a transmembrane glycoprotein with a molecular weight of 120 kDa involved in stem cells attachement to their niche, maintenance of cell polarity and migration through interactions of cells with each other and the matrix. This marker was originally identified as a marker of hematopoietic stem cells, but then its association with brain cancer, kidney, ovary, liver and colon cancers has been reported (1, 2, 10, 12 ). Despite the similarities among colon cancer stem cells, gastric stem cells have been studied less. Therefore, this study aimed to identify immunohistochemically cancer stem cells in gastric carcinoma and also to review the clinical and pathological features of patients.

## Materials and Methods

**Patients**

This research is a case- control study. The records of patients with gastric cancer who had undergone partial or total gastrectomy in Imam Khomeini Hospital, Sari, Iran from 2006 to 2012 were studied. None of the patients had received chemotherapy or radiotherapy before the surgery or presented other cancers at the same time.

**Immunohistochemical analyzes**

Formalin fixed paraffin-embedded tissues were extracted from the archives and proper well preserved sections from tumor and non- tumor regions (normal mucosa as a control tissue) were prepared for hematoxylin and eosin staining For immunohistochemical study, 4-micron-thick sec-tions were prepared and placed at 60°C for 1 h. For deparaffinization, the slides were placed in xylol solution (3 times), absolute ethanol and ethanol 96°, respectively (twice, 5 min each time), then washed and placed in running water. The slides were buffered in Tris- EDTA (pH= 9) and then placed in microwave at full power until the buffer reached the boiling point. After that, the microwave power was reduced to 40°C and all tissues stayed there for 15 min. Then slides were removed and placed at room temperature for 15 minutes. After rinsing under the running water and TBS buffer,slides were transfered into a damp chamber where murine monoclonal antibody diagnostic kits for CD44 (Abcam,DF1485, 1.200) and CD133 (Abcam, 27699,1.100) were applied on them respectively. Antibodies were diluted by diluant and carefully covered all tissue sections. Control tissues were covered just with TBS buffer and samples were placed at room temperature for 30 minutes. After washing with TBS buffer, hematoxylin was used as background stain. All IHC stained slides were observed by two expert pathologists (with no knowledge of patient's clinical data) and reported. The relationships between the expression of these markers and clinicopathologic features including age, sex, tumor size, histologic type of gastric adenocarcinoma, depth of tumor invasion, lymph node metastasis, lymphatic and vascular invasion were investigated. Membranous or cytoplasmic expression of the CD44 in more than 5 % of tumor cells, were considered as positive (7).

Membranous and cytoplasmic staining of CD133 in more than 10 % of tumor cells, were considered as positive (1).

**Statistical analyzes**

Data analysis were done using SPSS software (IBM SPSS Statistics 20.0.1) via X^2^, Fisher's exact test, Student's t- test and paired t- test to compare CD44 and CD133 expression in tumoral and non tumoral slides. A p-value below 0.05 was considered as statistically significant.

## Results

In this study, samples from 95 patients including 67 men (70.5%) and 28 females (29.5%) were enrolled and analyzed. The mean age of patients was 66.4± 1.15 years with a minimum and maximum age of 37 and 87 years, respectively. 37 patients (38.9% ) were < 65 years and 58 patients (61.1%) were > 65 years. 81 tumors were of intestinal type (85.3%) and 14 of diffuse type (14.7%). 23 of them were well (24.2%), 53 (55.8%) were moderately and 19 cases (20%) were poorly differentiated. 22 cases (23.25%) showed lymph node invasion, 9 (9.5%) had vascular invasion, 29 (30.5%) had both vascular and lymphatic invasion and 35 cases (36.8%) were non-invasive. The mean tumor size was 5.01± 0.23 cm. 42.1% (40 cases) were smaller than 4 cm, 47.4% (45 cases) were 4 to 8 cm and 10.5 % (10 patients) were larger than 8 cm. 37 cases of these samples had extended into serous and 28 of them had extended into muscularis mucosa.

**Fig. 1:**
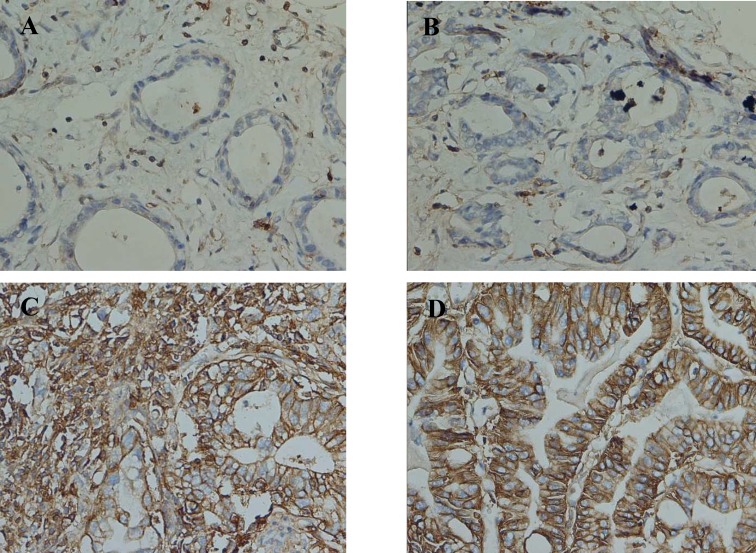
Immunohistochemical staining of gastric adenocarcinoma. CD44 negative control (A), CD133 negative control (B), CD44 and CD133 expressions in cytoplasm of tumor cells (C and D respectively). IHC (400 x)

In addition, the transition of tumor and non- tumor tissue of each sample (as control) was examined for the presence of CD44 and CD133. Expression of CD44 was not observed in the non-tumor areas, whereas CD133 was positive in 17 non– neoplastic samples. CD44 was positive in 57 tumor samples (60 %) (Figure 1C) whereas CD133 was positive in 47 tumor samples (49.5%) (Figure 1D). Frequencies of CD44 and CD133 positive and negative expression in tumor and non- tumor samples are described in table 1. Coexpression of CD44 and CD133 in gastric cancer samples displayed a positive correlation using t-test and Fisher's exact test.

**Table 1:** Frequece of CD44 and CD133 IHC markers in Gastric adenocarcinoma 81234567890

**CD44**	**CD133**		
	Tumor /+Normal +	Tumor /+ Normal -	Tumor- /Normal -
**Tumor** ** + / ** **Normal ** ** -**	8 (14%)	14 (24.6%)	35 (61.4%)
**Tumor** ** - / ** **Normal ** ** -**	9 (23.7%)	16 (1.34%)	13 (34.2%)
X^2^ = 6.74 P=0.034			

Tumor + : expression of the marker in tumor, Tumor-: no expression of the marker in tumor, Normal+: expression of the marker in non tumoral tissue, Normal-: no expression of the marker in non tumoral tissue

**Table 2:** Frequency of CD44 expression related to age and tumor characteristics in gastric adenocarcinoma

		Tumor +/Normal -	Tumor -/Normal -	p value
Age	< 65	(43.9%) 25	(31.6%) 12	0.354
	≥ 65	(56.1%) 32	(68.4%) 26	
Type of cancer	Intestinal	(89.5%) 51	(8.9%) 3	< 0.001
	Diffuse	(10.5%) 6	(21.2%) 8	
Size	< 4cm	(47.4%) 27	(34.2%) 13	0.003
	4-8 cm	(40.4%) 23	(57.9%) 22	
	> 8cm	(12.3%) 7	(7.9%) 3	
Depth of invasion	Serosa	(71.9%) 41	(68.7%) 26	0.001
Vascular invasion	Lymphatic invasion	(24.6%) 14	(21.1%) 8	0.009
	Vascular invasion	(7.0%) 4	(13.2%) 5	
	Vascular and Lymphatic invasion	(31.6%) 18	(28.9%) 11	
	Without invasion	(36.8%) 21	(32.8%) 17	
Tumor grade	Well	(23.5%) 12	(34.7%) 11	< 0.001
	Moderate	(72.5%) 37	(53.3%) 16	
	Poor	(3.9%) 2	(10.0%) 3	

CD44 expression frequency in tumor and non- tumor samples according to age, size, type and invasion of tumor is described in Table 2. Expression of this stem cell marker was associated to intestinal type of gastric cancer, tumor size less than 8 cm (4- 8cm), serous invasion without lymphatic and vascular invasion, and had significant relationship with moderately differentiated tumors as 72.5 % of the specimens were positive for this marker (Table-2).

Distribution of CD133 expression in tumor and non- tumor samples based on age, size, type and invasion of tumor was fully described in Table 3. As it stands to reason, CD133 expression showed positive correlation with age over 65 years, intestinal type gastric cancer, size 4 to 8 cm, moderately differentiated tumor and serous involvement.

## Discussion

The annual incidence of gastric cancer is 2 % of all new cancers in the United States and it is also common in many other countries, particularly in East Asia and South America (13). Rocco et al. studied 47 gastric carcinomas in which CD133 and CD44 were positive in most tumor cells, but there was not any cancerous lesion after their transplantation into immunocompromised mice (2).

In another study, Philipilidis et al. claimed that Helicobacter pylori and stem cells signaling may be associated with tumor initiation and progression (14, 15). Sadhna Dhingra et al. proposed that stemcells may result in intestinal metaplasia or gastric adenocarcinoma, intestinal type (16). Moreover, Lee et al. noticed that resistance to chemotherapy and recurrence of tumor can be caused by CD133 (17).

**Table 3:** Frequency of CD133 expression related to age and tumor characteristics in gastric adenocarcinoma

		Tumor +/Normal +	Tumor +/Normal -	Tumor-/Normal -	P value
Age	< 65	(29.4%)5	(20%)6	(54.2%)26	<0.001
	≥ 65	(70.6%)12	(80%)24	(45.8%)22	
Type of cancer	Intestinal	(82.4%)14	(83.3%)28	(81.3%)39	<0.001
	Diffuse	(17.6%)3	(6.7%)2	(18.8%)9	
Size	< 4cm	(53.3%)6	(30%)9	(53.1%)25	<0.001
	4-8cm	(58.8%)10	(60%)18	(35.4%)17	
	> 8cm	(5.9%)1	(10%)3	(12.5%)6	
Depth of invasion	Serosa	(76.5%)13	(68.7%)26	(68.3%)28	<0.001
Vascular invasion	Lymphatic invasion	(17.6%)3	(26.7%)8	(23.9%)11	0.34
	Vascular invasion	(11.8%)2	(16.7%)5	(40.2%)2	
	Vascular and lymphatic invasion	(41.2%)7	(26.7%)8	(29.2%)14	
	Without invasion	(29.4%)5	(30%)9	(43.8%)21	
Tumor grade	Well	(7.1%)1	(10.7%)3	(48.7%)19	< 0.001
	Moderate	(71.4%)10	(85.7%)24	(48.7%)19	
	Poor	(21.4%)3	(3.6%)1	(2.6%)1	

Several experimental studies on animal models (rats) have shown that cancer stem cells could play an important role in the initiation and progression of gastric cancer, as in other gastrointestinal tumors (18-22). Takaishi et al. were able to demonstrate cancer stem cells in human gastric cancer by CD44 marker (19). Heider et al. have shown the presence of CD44 isomers in gastric cancer and their association with pathologic and clinical aspects of gastric cancer, since 1993. This molecule was originally identified as a lymphocyte- homing receptor (23). CD44 is a member of cell adhesion receptor family and its gene which has several isomers is located on chromosome 11p12-13. The standard isomer of CD44 is expressed predominantly in normal tissues (24). Ishimito et al. also demonstrated the presence of "stem– like, slow cycle cells" expressing CD44 in gastric glands at the squamo- columnar junction in normal mice and highlighted the role of these cells in the development of lethal cancers of the stomach (25).

In our study, the frequency of CD44 positive cells in tumor samples was 60 % and all non- tumoral samples were negative. In the study of Dhimgra et al. the incidence of the CD44 expressing cells was 51% in tumor and 15% in non- tumoral tissue (16). Ghafarzadegan et al. also evaluated the incidence of CD44 in 100 cases of gastric cancers and found that the incidence of CD44 expression was 65 % (11) which was compatible with our result. Kim et al. studied 211 cases of stomach cancer and demonstrated that the incidence of CD44 was 11.4% for s- isomer, but they did not find the V6 isomer (26). Yamagachi et al. in a study of 95 cases of gastric carcinoma reported that the expression of CD44 v6 was 47.3% as well (27). Different frequencies of CD44^+^ cells in gastric carcinomas reported in various studies could be due to geographic variation and various CD44 antibodies were used to identify isomers. In our study, the expression of CD44 in intestinal type gastric cancer was significantly higher than the diffuse type and it was interestingly associated with the tumor size (4-8 cm), depth (serous involvement) and grade (moderately differentiated). It also had an inverse relationship with the extent of invasion in a way that the highest expression was in tumors without invasion. However, Dhimgra et al. found that the expression of CD44 in the intestinal type gastric cancer was also high and was not correlated with other pathologic and clinical parameters (16). In the study of Kim et al. the expression of CD44 was shown to be related to tumor size and stage, survival, vascular invasion, lymph node metastases and perineural invasion as in our study (26). Similarly, Ghafarzadegan et al. presented statistically significant association between the expression of CD44 with histological grade and survival(11). The relationship between the incidence of CD44 and survival rate of metastatic spread has been reported in several studies (1, 27, 28). Dammrich et al. found that the CD44 (v6 isomer) was expressed in the chronic atrophic gastritis and intestinal metaplasia with dysplastic change (28). As the metaplastic glands with dysplastic change are precancerous lesions of gastric carcinoma, so the expression of stem cell markers represents the epigenetic changes and supports the role of stem cells in intestinal- type gastric carcinoma (11, 26-29).

CD133 is the other marker of cancer stem cells that is expressed in more than 50% of cells in many malignancies, including gastric and pancreatic cancers and intrahepatic cholangioca-rcinoma (12). However, the expression of CD133 is not restricted to stem cells and the metastatic colon cancer cells can be the origin of new tumor growth independently of the presense of CD133 (30, 31). It has been shown that anti-CD133 antibodies could effectively inhibit cancer cell growth and induce apoptosis in gastric cancer cells *in vitro* (12). Zhao et al. showed that the expression of CD133 in gastric cancer samples was 57.4% and a close association of CD133 with poor prognosis in gastric adenocarcinoma was identified (1). Basically, studies related to the incidence and prognostic significance of CD133 in gastric cancer were limited and inadequate. Thus in this study, intentionally CD133 was examined and it was shown that it might be related to tumor size, depth and histologic grade (1), which correlated with previous reports concerning the role of CD133 in colorectal cancer (32, 33). Lee H et al. also reported about 23% expression of CD133 in gastric cancer which was significantly related to serous involvement, venous invasion and well to moderate differentiation in intestinal type gastric cancer. They also reported that 5- year survival rate in patients with CD133 expression was worse than in negative patients (17). In our study, the expression of CD133 in tumor samples was 49.5%. It was also positive in 17 (31.6%) non-tumoral tissues. This marker's expression was higher in participants over 65 years age and showed significant association with serous involvement, tumor size and differentiation, but unlike some of the previous studies was not significantly associated with vascular or lymphatic invasion. Zhao et al. showed that the CD133 positivity had a good relationship with lower survival rate (1). They suggested that CD133^+^ cells including stem cell-like cells might lead to recurrences and metastases which is consistent with the hypothesis of cancer stem cells (1, 17, 34, 35). According to our findings, co- expression of CD44 and CD133 had also a positive correlation with clinical data as shown in Table 2. This association was not investigated in previous studies. Further studies may be needed to confirm this association.
